# Overview of the Nontyphoidal and Paratyphoidal *Salmonella* Vaccine Pipeline: Current Status and Future Prospects

**DOI:** 10.1093/cid/ciaa514

**Published:** 2020-07-29

**Authors:** Scott M Baliban, Ying-Jie Lu, Richard Malley

**Affiliations:** 1 Center for Vaccine Development and Global Health, University of Maryland, School of Medicine, Baltimore, Maryland, USA; 2 Division of Infectious Diseases, Boston Children’s Hospital, Harvard Medical School, Boston, Massachusetts, USA

**Keywords:** *Salmonella*, NTS, Paratyphi, vaccines

## Abstract

Nontyphoidal *Salmonella* and *Salmonella* Paratyphi are responsible for significant morbidity and mortality worldwide. To date, no vaccine has been licensed against these organisms. The development of effective vaccines remains an urgent priority. In this review, the rationale for and current status of various vaccine candidates against *S*. Paratyphi and nontyphoidal *Salmonella* are presented, with a focus on the research findings from the 2019 International Conference on Typhoid and Other Invasive Salmonelloses. Additionally, other vaccine candidates that are currently undergoing clinical development are highlighted. Future approaches, which may include antigens that are genetically conserved across *Salmonella* and confer broad, non–serotype-specific protection, are also discussed.

Enteric fever is a major cause of morbidity and mortality in the developing world. The 2 major pathogens responsible for enteric fever are *Salmonella enterica* serovar Typhi (*S*. Typhi) and *S*. Paratyphi A. These organisms cause acute illnesses characterized by fevers, chills, abdominal pain, and in the more severe cases, hemodynamic compromise. While, in general, Paratyphi strains tend to have a more indolent course, both *S*. Typhi and *S*. Paratyphi (including Paratyphi A and B) can cause life-threatening illness. Incidence rates of paratyphoid disease vary widely according to geography. Estimates of age-standardized incidence rates of combined typhoid and paratyphoid disease range from 549 cases per 100 000 person-years in South Asia, 81 in central sub-Saharan Africa, and 39 in North Africa and the Middle East, with paratyphoid disease accounting for approximately 25% of these cases [1].

In sub-Saharan Africa, nontyphoidal *Salmonella* serovars Enteritidis and Typhimurium (including monophasic variant 1,4,[5],12:i:−) are a major pediatric public health concern. The bulk of invasive nontyphoidal *Salmonella* (iNTS) disease (typified by bacteremia, septicemia, meningitis) occurs in children under 5 years old, and case-fatality rates range from 12% to 28%. Invasive nontyphoidal *Salmonella* infections are widespread and reported as a cause of bacteremia in 33 out of 54 African countries. Furthermore, the rise in antibiotic resistance makes the treatment of these infections increasingly problematic [2, 3].

At present, there is no paratyphoid or iNTS vaccine licensed for use in humans, and development of effective interventions, including vaccination, is needed.

## VACCINE CANDIDATE PIPELINE AND STATUS

### Live-attenuated Approaches

#### Salmonella Paratyphi A 

A few live-attenuated *S*. Paratyphi strains have been developed and tested in animal models [4–6]. The University of Maryland’s Center for Vaccine Development and Global Health (CVD) generated CVD 1902 by introducing 2 deletions in the guaBA operon (which is required for the biosynthesis of guanine nucleotides) and the clpX operon (which encodes a chaperone ATPase) of *S*. Paratyphi A strain ATCC 9150 [6]. This vaccine candidate was shown to be safe and protective in a murine intraperitoneal challenge model with wild-type *S*. Paratyphi A. The results of a phase I clinical trial that evaluated T-cell–mediated immunity elicited in volunteers following immunization with CVD 1902 [7] were presented at the 2019 International Conference on Typhoid and Other Invasive Salmonelloses. A single dose of either 10^9^ colony forming units (CFU) or 10^10^ CFU of CVD 1902 elicited *S*. Paratyphi A–specific memory CD8^+^ and CD4^+^ T-cell responses in 7 of 12 of the volunteers receiving CVD 1902 compared with 0 of 4 volunteers who received placebo. Although the number of subjects was small and the latest time point at which T cells were detected was short (day 28), these results are encouraging as they indicate that CVD 1902 is immunogenic in humans and can induce CD4^+^ and CD8^+^ T-cell responses. CVD 1902 has been licensed to Bharat Biotech International, Ltd (Hyderabad, India), which has also licensed the live oral *S*. Typhi vaccine strain, CVD 909. These developments provide some hope that a future bivalent live oral vaccine that targets these 2 important pathogens may be developed commercially in the future.

#### Salmonella Paratyphi B.

Currently, there are very few descriptions of live-attenuated *S.* Paratyphi B vaccine candidates. An *S*. Paratyphi B mutant was generated by introducing 2 known attenuating mutations, ∆*guaBA* and ∆*clpX*, into *S*. Paratyphi B *sensu stricto* (strain CMF 6999), creating vaccine strain CVD 2005 [8]. These 2 mutations have previously been used in vaccines for other *Salmonella* serovars, including Typhimurium and Enteritidis [9] and Paratyphi A [6]. Immunization with CVD 2005 in mice by both oral and intranasal routes generated immunoglobulin (Ig) G (IgG) antibody against *S*. Paratyphi B lipopolysaccharide (LPS), and sera from immunized mice showed bactericidal and opsonophagocytic killing activity. Furthermore, immunized mice showed 90% protective efficacy following challenge with a homologous strain and 42% efficacy in a heterologous challenge.

#### Nontyphoidal Salmonella 

A number of live-attenuated iNTS vaccine candidates have been evaluated preclinically [10, 11]; however, to date, only 2 clinical studies have been conducted in humans. In the first, researchers at the Massachusetts General Hospital in Boston engineered *S. Typhimurium* strain LH1160, a chicken isolate defective in purine synthesis (∆*purB*) and *Salmonella* Pathogenicity Island (SPI)-2 gene expression (∆*phoP/Q*). LH1120 was given to healthy volunteers as a single oral dose of 5–8 × 10^7^ CFU, which resulted in IgG responses to either LPS or flagellar antigens in 5 of 6 subjects. Mild reactogenicity (acute onset of fever and constitutional symptoms) was noted in 2 subjects, and no instance of bacteremia was detected. Fifty percent of volunteers had positive stool cultures for up to 10 days following administration, after which point they were treated with an oral antibiotic to ensure clearance [12]. In the second trial, a human *S.* Typhimurium gastroenteritis isolate from the United Kingdom (strain WT05; Microscience) was attenuated by mutations of a gene involved in aromatic amino acid synthesis (∆*aroC*) and SPI-2 effector protein secretion (∆*ssaV*). Eighteen healthy adult volunteers were immunized orally with a single dose of WT05 ranging from 10^7^ to 10^9^ CFU. While WT05 was well tolerated, the LPS-specific serum IgA and IgG responses were highly variable, and persistent shedding of bacteria for up to 12–23 days was noted [13].

### Glycoconjugates

#### Nontyphoidal Salmonella Core and O-polysaccharide:Flagellin. 

The *Salmonella* glycoconjugate approach is geared towards generating a protective antibody response against the surface polysaccharide, which is the core and O-polysaccharide (COPS) in NTS and paratyphoid serovars. Covalent linkage of bacterial polysaccharides to protein carriers engages CD4^+^ T-cell help and dramatically enhances polysaccharide immunogenicity. Several conjugation strategies have been explored and shown to offer protection against *Salmonella* in rodents (summarized in [10]). Researchers at the CVD have developed COPS:FliC glycoconjugates, where the phase I flagellin monomer (FliC) from the homologous serovar acts as both the carrier protein and a secondary antigenic target. Simple, robust, and readily scalable processes for the purification of Good Manufacturing Practice (GMP)–grade COPS and FliC have been developed [14, 15]. The conjugation process effectively ablates the Toll-like receptor (TLR) 5–stimulating activity of flagellin monomers, which may increase the safety profile of these candidate vaccines [15]. COPS can be linked to FliC using different chemistries and linkers; however, to maintain native O-acetylation patterns on the O-antigen, it is necessary to utilize a neutral pH chemistry [16].

Baliban et al [17] presented the preclinical development of S. Enteritidis and S. Typhimurium COPS:FliC conjugates. S. Enteritidis COPS:FliC was robustly immunogenic in both adult and infant mice and protective against lethal systemic challenge with virulent Malian blood isolates. S. Enteritidis and S. Typhimurium COPS:FliC were further tested in rabbits where they were co-formulated with Typbar-TCV (Vi:tetanus toxoid [TT], manufactured by Bharat Biotech) as a trivalent typhoid-iNTS glycoconjugate vaccine. Immunized rabbits generated high IgG titers to all polysaccharides and the FliC carrier antigens. Equivalent anti-COPS responses were obtained in monovalent and multivalent formulations. Rabbit sera demonstrated functional antibacterial activity in vitro and passively protected mice against fatal challenge [18]. The COPS:FliC technology has been licensed to Bharat Biotech, and a first-in-human clinical trial for the trivalent typhoid-iNTS conjugate vaccine is anticipated in the near future.

#### Paratyphoidal Salmonella OPS-based Conjugates. 

*Salmonella* Paratyphi A OPS:TT conjugates were previously shown to be safe and to elicit anti-OPS IgG antibodies in phase I and II studies in adults, teenagers, and 2- to 4-year-old children [19]. O-polysaccharide conjugates made with other carriers (eg, diphtheria toxoid and cross-reacting material [CRM]_197_) have been tested in animal models and shown to be immunogenic (summarized in [20]). More recently, Sun et al [21] used an in vivo glycosylation system in a modified *S*. Paratyphi A strain to generate a conjugate that was subsequently shown to be immunogenic and generate functional antibodies in mice.

### Multiple Antigen Presenting System Complexes

The Multiple Antigen Presenting System (MAPS) is a promising alternative to traditional conjugation approaches [22–24]. This novel technology uses the affinity pair biotin-rhizavidin to generate a complex of polysaccharide and proteins. The MAPS technology is a highly efficient way of enhancing the immunogenicity of polysaccharides. MAPS-based vaccines induce robust, boostable, and CD4^+^ T-cell–dependent anti-polysaccharide antibody responses, as well as functional antibody and T-helper (Th) 1/Th17cell responses to carrier proteins. Lu and Malley [25] presented their findings using a bivalent vaccine against *S*. Typhi Vi and *S*. Paratyphi A OPS MAPS. The presence of Vi-specific memory B cells was confirmed by adoptive transfer experiments in Rag-deficient mice. Three different carrier proteins were compared, and a pneumococcal fusion protein was selected to construct both Vi and OPS MAPS. Dosing of polysaccharides were tested in several animal models (including mice, rabbits, and guinea pigs). Postimmunization, the presence of functional antibodies directed against Vi and OPS was confirmed using bactericidal and opsonophagocytic killing assays. Avidity analysis of postimmunization rabbit sera also confirmed affinity maturation for both Vi and OPS MAPS. Thus, these preclinical results strongly support the feasibility of this approach and the potential to apply this technology to other important *Salmonella* serovars. This work is currently being pursued at Boston Children’s Hospital, with support from the Bill and Melinda Gates Foundation.

### Generalized Modules for Membrane Antigens

Outer membrane vesicles (OMVs) are spontaneously secreted by many gram-negative bacteria and represent an attractive vaccine platform as they are nonreplicating and theoretically display similar antigen density and pathogen-associated molecular patterns (and therefore immunostimulatory profiles) as their live parental strains. The GSK Vaccines Institute for Global Health (GVGH) has developed a low-cost, scalable, and standardized manufacturing system for producing GMP-quality OMVs, referred to as Generalized Modules for Membrane Antigens (GMMA). Targeted disruption of the Tol-Pal apparatus (∆*tolR*), which supports outer membrane integrity, leads to overproduction of GMMA with a consistent diameter (20–110 nm) [26]. Further deletion of *pagB* and *msbB* results in predominantly penta-acylated lipid A structures, which afford GMMA with a lower proinflammatory profile in vitro [27].

Koeberling et al [28, 29] presented the preclinical development of a novel *S. Typhimurium* GMMA, as part of a 2-component *S. Enteritidis*/*S. Typhimurium* GMMA vaccine. Immunization of mice and rabbits with *S. Typhimurium* GMMA elicited robust and boostable anti-OPS IgG responses. A comparison of subcutaneous and intranasal immunization routes revealed that anti-OPS serum IgG and intestinal IgA titers were highest after intranasal GMMA delivery. Whereas intranasal immunization skewed the anti-OPS IgG response towards Th1, subcutaneous delivery yielded a balanced Th1/Th2 profile. Finally, multifunctional Th1/Th17 CD4^+^ T cells were observed after both subcutaneous and intranasal immunization. A phase I immunogenicity and safety study for *S. Enteritidis* and *S. Typhimurium* GMMA in healthy adult volunteers is being planned.

## POTENTIAL FUTURE DIRECTIONS

*Salmonella* Paratyphi A (serogroup A), *S*. Typhimurium (serogroup B), and *S*. Enteritidis (serogroup D) are well appreciated as the leading causes of paratyphoid fever and iNTS disease. While the vaccines described above offer much promise for the eventual control of *Salmonella* disease, an open question is whether the current vaccine pipeline can offer cross-protective immunity against nonvaccine serovars and mitigate potential serotype replacement after large-scale introduction. Of interest is serogroup C *Salmonella* whose burden is underestimated and increasing worldwide [30, 31]. Taking advantage of widely conserved *Salmonella* antigens may offer one potential solution to increase the cross-reactivity of vaccine candidates. Outer membrane proteins (eg, OmpC, OmpD, OmpF) [32, 33], siderophores (enterobactin) [34], and type III secretion system proteins (eg, SipB, SipD, SseB, SseC, and PrgI) [35–37] have been explored as vaccine antigens in mice, demonstrating robust immunogenicity and protective efficacy against homologous strains. Alternatively, access to conserved membrane antigens can be enhanced for live-attenuated and OMV platforms. As an example, Liu et al reported the utility of OMVs derived from *S*. Typhimurium mutants lacking flagella [38] or expressing truncated LPS [39] to promote cross-protective immunity against both *S*. Enteritidis and *S*. Choleraesuis (serogroup C). Finally, multivalent approaches such as those discussed in this commentary, where heterologous *Salmonella* antigens are simultaneously expressed in a single vaccine platform, offer a practical and cost-effective strategy for targeting co-endemic serovars. Recent examples of this include live-attenuated *S*. Typhimurium strains engineered to express *S*. Paratyphi A OPS [40] or both *S*. Enteritidis OPS and *S*. Typhi Vi [41]. Mice that were immunized with these recombinant strains developed a broadly bactericidal or opsonophagocytic antibody response [40, 41] and were protected from lethal challenge with either *S*. Enteritidis or *S*. Typhimurium [41].

## CONCLUSIONS

Infections caused by *Salmonella* Enteritidis, Typhimurium, and Paratyphi represent a significant and currently unmet public health concern. Several monovalent and multivalent vaccines currently in preclinical and clinical development were presented during the conference. Two first-in-human studies of candidate *S*. Enteritidis and *S*. Typhimurium COPS:FliC and GMMA vaccines are eagerly anticipated. More work is needed to ensure the availability of safe and efficacious *Salmonella* vaccines that are broadly protective. 

## Notes

***Supplement sponsorship.*** This was sponsored by the Bill and Melinda Gates Foundation (OPP1182032).

***Financial support.*** This supplement is funded with support from the Coalition against Typhoid Secretariat, housed at the Sabin Vaccine Institute in Washington, DC and made possible by a grant from the Bill & Melinda Gates Foundation.

***Potential conflicts of interest.*** Y.-J. L. is a scientific founder, consultant, and owner of equity of Affinivax, a biotechnology vaccine development company that is developing vaccines using the MAPS technology described in this article. R. M. is a scientific founder, member of the board of directors, consultant, and owner of equity of Affinivax. S. M. B. reports no potential conflicts. All authors have submitted the ICMJE Form for Disclosure of Potential Conflicts of Interest. Conflicts that the editors consider relevant to the content of the manuscript have been disclosed.
